# Socio-economic inequality of utilization of cancer testing in Europe: A cross-sectional study

**DOI:** 10.1016/j.pmedr.2022.101733

**Published:** 2022-02-08

**Authors:** H. Bozhar, M. McKee, T. Spadea, P. Veerus, S. Heinävaara, A. Anttila, C. Senore, N. Zielonke, I.M.C.M. de Kok, N.T. van Ravesteyn, I. Lansdorp-Vogelaar, H.J. de Koning, E.A.M. Heijnsdijk, on behalf of the EU-TOPIA consortium

**Affiliations:** a Erasmus MC, University Medical Center Rotterdam, Department of Public Health, Rotterdam, the Netherlands; b London School of Hygiene and Tropical Medicine, London, UK; c Epidemiology Unit, ASL TO3 Piedmont Region, Grugliasco (Turin), Italy; d National Institute for Health Development, Tallinn, Estonia; e Finnish Cancer Registry, Helsinki, Finland; f SC Epidemiology, Screening, Cancer Registry, Città della Salute e della Scienza University Hospital, CPO, Turin, Italy

**Keywords:** Inequality, Cancer screening, Socio-economic position, Europe

## Abstract

There are currently screening programmes for breast, cervical and colorectal cancer in many European countries. However, the uptake of cancer screening in general may vary within and between countries. The aim of this study is to assess the inequalities in testing utilization by socio-economic status and whether the amount of inequality varies across European regions. We conducted an analysis based on cross-sectional data from the second wave of the European Health Interview Survey from 2013 to 2015. We analysed the use of breast, cervical, and colorectal cancer testing by socio-economic position (household income, educational level and employment status), socio-demographic factors, self-perceived health and smoking behaviour, by using multinomial logistic models, and inequality measurement based on the Slope index of inequality (SII) and Relative index of inequality (RII). The results show that the utilization of mammography (Odds Ratio (OR) = 0.55, 95% confidence interval (95%CI):0.50–0.61), cervical smear tests (OR = 0.60, 95%CI:0.56–0.65) and colorectal testing (OR = 0.82, 95%CI:0.78–0.86) was overall less likely among individuals within a low household income compared to a high household income. Also, individuals with a non-EU country of birth, low educational level and being unemployed (or retired) were overall less likely to be tested. The income-based inequality in breast (SII = 0.191;RII = 1.260) and colorectal testing utilization (SII = 0.161;RII = 1.487) was the greatest in Southern Europe. For cervical smears, this inequality was greatest in Eastern Europe (SII = 0.122;RII = 1.195). We concluded that there is considerable inequality in the use of cancer tests in Europe, with inequalities associated with household income, educational level, employment status, and country of birth.

## 1 Introduction

Breast, colorectal and cervical cancer are contributing substantially to the overall cancer burden in Europe, with more than 1 million new cases, and 407,000 deaths in 40 European countries in 2018 (Ferlay et al., 2018). Although detection and treatment of cancer have improved considerably in recent decades, the death rate remains high (Ferlay et al., 2018). Moreover, the cancer burden is unequally distributed within and among countries, with differences in risk, uptake of screening, and access to treatment (Aarts et al., 2012, Deandrea et al., 2010, Palencia et al., 2010, Puliti et al., 2012, Cardoso et al., 2020, Mahumud et al., 2020, Nunes et al., 2021, Pallesen et al., 2021). These differences all are associated with socioeconomic position. For example, people having a low socioeconomic position have a higher risk of developing cervical and colorectal cancer (Braaten et al., 2005, Hastert et al., 2015). On the contrary, women with a high socio-economic position have a higher risk of developing breast cancer, thought to reflect nutrition in childhood, reproductive history, and exposure to hormonal therapies (Lundqvist et al., 2016). Individuals who have a lower socio-economic position, or a lower education level tend to be diagnosed at a more advanced stage of cancer and experience worse survival rates, partly as a result of lower screening participation (Puliti et al., 2012, Aarts et al., 2011, Hoeck et al., 2019, Artama et al., 2016, Reducing social inequalities in cancer: evidence and priorities for research. International Agency for Research on Cancer, 2019).

Cancer burden is also unequally distributed across Europe. Age-standardised mortality rates of breast, cervical and colorectal cancer are 2–3 fold higher in Eastern European countries than in Western European countries (Bertuccio et al., 2019). These differences in cancer mortality rates are caused by differences in treatment and in the effectiveness of screening programmes. Most countries in Europe have organised screening programmes for breast, cervical and colorectal cancer. However, screening programmes are implemented in different ways, with consequences for levels of participation by different groups in societies (Deandrea et al., 2010, Palencia et al., 2010). Also opportunistic testing is still common, especially in Eastern Europe. Well-managed population-based screening programmes can achieve more equitable access than opportunistic testing (Palencia et al., 2010, Puliti et al., 2012, Andermann et al., 2008, Pacelli et al., 2014, Zengarini et al., 2016).

The aim of this study is to assess the scale and nature of social inequality in the use of services for early detection of cancer according to demographic and socioeconomic characteristics. In addition, the difference in inequality between European regions is quantified, which can contribute to highlighting this issue on the policy agenda for the countries concerned. The present study focused on the use of various tests for the three most common cancer types: mammography, cervical smear, and faecal occult blood (FOB) or colonoscopy testing.

## 2 Methods

To evaluate existing inequalities in European cancer screening, we performed a cross-sectional analysis of the association between selected socio-economic variables and the use of tests.

The individual level data that we used were obtained from the second wave of the European Health Interview Survey (EHIS). The EHIS survey is conducted approximately every five years, among people of at least 15 years old, living in a private household. All of the then 28 EU countries (listed in the footnote of Table 1 plus Norway and Iceland participated in the second wave in the period 2013–2015 (European Health Interview Survey (EHIS)). Ethical approval for the study was provided by the European commission. This study met the guidelines for protection of human subjects concerning their safety and privacy. EHIS covers approximately 340 variables including demographic characteristics (i.e. age, gender, country of birth, citizenship), socio-economic factors, healthcare utilization, health status (i.e. chronic illness, disabilities) and health determinants. The questionnaire contains 130 questions and the main elements of interest for this study were the use of various tests to detect cancer and the socio-economic factors including household income (European quintiles, since country quintiles were not available), highest attained educational level (based on the International Standard Classification of Education 2011; low: until lower secondary education, intermediate: upper secondary education to post-secondary, high: tertiary education) and employment status. All covariates were a priori selected based on literature, assuming that the following factors can be related to both socioeconomic position and health seeking behaviour like screening participation. For confounders, sociodemographic variables like age, gender, country of birth, European region of residence (North, East, South, West), urbanization and marital status were included. Finally, other variables included in this study were self-perceived health to account for general health and smoking behaviour as lifestyle indicator. The response rates varied by country, ranging from 30% to about 84%. Seventeen countries had a response rate exceeding 60%. We excluded data of Malta and Iceland because some key variables for this analysis were missing or categorized differently.

**Table 1:** Characteristics of the EHIS sample by cancer screening type.

	Mammography testing (50–69 aged females) N = 56,807	Cervical smear testing (30–64 aged females) N = 95,352	Colorectal testing (age 50–74) N = 125,239
			*Males: 57,451 (45.9%)* *Females: 67,788 (54.1%)*

Variable	N	%	N	%	N	%
*Last test*						
Not applicable ^a^			252	0.3		
Never	7281	12.8	10,080	10.6	61,881	49.4
Not up to date ^b^	12,038	21.2	14,186	14.9	12,337	9.9
Up to date ^c^	36,088	63.5	68,026	71.3	47,244	37.7
Missing	1400	2.5	2808	2.9	3777	3.0
*Age*						
30–34			11,181	11.7		
35–39			12,650	13.3		
40–44			13,824	14.5		
45–49			14,426	15.1		
50–54	14,557	25.6	14,557	15.3	27,269	21.8
55–59	14,469	25.5	14,469	15.2	26,760	21.4
60–64	14,245	25.1	14,245	14.9	26,355	21.0
65–69	13,536	23.8			24,940	19.9
70–74					19,915	15.9

*European region of residence*^d^						
Western Europe	18,142	31.9	31,240	32.8	40,318	32.2
Eastern Europe	13,397	23.6	21,604	22.7	29,012	23.2
Southern Europe	14,890	26.2	25,739	27.0	33,063	26.4
Northern Europe	10,378	18.3	16,769	17.6	22,846	18.2

*Country of birth*						
Native-born	52,075	91.7	84,930	89.1	115,246	92.0
Non-EU country	2602	4.6	5777	6.1	5342	4.3
Other EU state	1762	3.1	3965	4.2	3758	3.0
Missing	368	0.6	680	0.7	893	0.7

*Urbanization*						
Densely-populated	19,661	34.6	33,379	35.0	42,319	33.8
Intermediate-populated	16,966	29.9	28,699	30.1	37,171	29.7
Thinly-populated	20,118	35.4	33,173	34.8	45,605	36.4
Missing	62	0.1	101	0.1	144	0.1

*Marital status*						
Married	37,321	65.7	62,821	65.9	85,611	68.4
Divorced	7440	13.1	11,025	11.6	14,255	11.4
Widowed	7251	12.8	4945	5.2	13,662	10.9
Never married	4679	8.2	16,365	17.2	11,439	9.1
Missing	116	0.2	196	0.2	272	0.2

*Educational level*						
High	12,679	22.3	30,689	32.2	27,677	22.1
Intermediate	35,079	61.8	56,373	59.1	75,981	60.7
Low	8681	15.3	7671	8.0	20,749	16.6
Missing	368	0.6	619	0.6	832	0.7

*Employment status*						
Working	23,309	41.0	59,655	62.6	48,786	39.0
Other	8037	14.1	15,078	15.8	10,953	8.7
Permanently disabled	2346	4.1	3047	3.2	5025	4.0
In (early) retirement	19,910	35.0	9371	9.8	53,547	42.8
Unemployed	2865	5.0	7633	8.0	6241	5.0
Missing	340	0.6	568	0.6	687	0.5

*Household monthly income quintiles*						
Between 4th-5th quintile	11,268	19.8	20,018	21.0	25,252	20.2
Between 3rd-4th quintile	11,037	19.4	19,493	20.4	24,417	19.5
Between 2nd-3rd quintile	10,877	19.1	17,381	18.2	24,115	19.3
Between 1st and 2nd quintile	10,403	18.3	15,982	16.8	23,181	18.5
Below 1st quintile	9493	16.7	16,383	17.2	20,209	16.1
Missing	3729	6.6	6095	6.4	8065	6.4

*Self-perceived health*						
Very good	7381	13.0	20,457	21.5	15,686	12.5
Good	23,861	42.0	45,220	47.4	52,453	41.9
Fair	18,398	32.4	21,579	22.6	40,122	32.0
Bad	4942	8.7	5044	5.3	11,339	9.1
Very Bad	1129	2.0	1064	1.1	2614	2.1
Missing	1096	1.9	1988	2.1	3025	2.4

*Smoking behaviour*						
No smoking	45,185	79.5	72,191	75.7	96,912	77.4
Occasional smoking	1779	3.1	4109	4.3	4119	3.3
Daily smoking	9088	16.0	17,781	18.6	22,486	18.0
Missing	755	1.3	1271	1.3	1722	1.4

a Not applicable because of hysterectomy.

b Not up to date = received mammography more than 2 years ago, cervical smear test more than 3 years ago, or received FOB-test more than two years ago and/or received colonoscopy more than ten years ago.

c Up to date = received mammography within past 2 years, cervical smear test within past 3 years, or received FOB-test within past two years and/or received colonoscopy within past ten years.

d Northern Europe: Denmark, Estonia, Finland, Ireland, Lithuania, Latvia, Norway, Sweden.

Western Europe: Austria, Belgium, Germany, France, Luxembourg, Netherlands, United Kingdom.

Eastern Europe: Bulgaria, Czech Republic, Hungary, Poland, Romania, Slovenia, Slovakia.

Southern Europe: Cyprus, Greece, Spain, Croatia, Italy, Portugal.

### 2.1 Primary outcome variables

The main outcome variables captured utilization of services for early detection of cancer. Respondents were asked when they last received mammography (never/more than 2 years/within the past 2 years) and what was the last time of having a cervical smear test (never/more than 3 years/within the past 3 years). Respondents were also asked about their last FOB-test (never/more than 2 years/within the past 2 years), and the last time they had colonoscopy (never/more than 10 years/within the past 10 years). We combined the last two variables into one, representing whether individuals were overall up to date with colorectal cancer screening (never screened/not up to date/up to date). Individuals who either received a FOB-test within the past two years or a colonoscopy within the past ten years were considered to be up to date with colorectal cancer screening. These responses either indicate participation in a screening programme or the use of testing services outside the screening programmes, or for diagnostic purposes, since there was no data about the reason for having the test.

### 2.2 Statistical analysis

For every analysis conducted within this study, we included respondents based on gender and age (50–69, 30–64, and 50–74 years) to define those eligible for screening programmes for breast, cervical and colorectal cancer, respectively, since this is the minimal age range in most countries.

We imputed missing values (varying between 0% and 6.4% per question) using a multiple imputation method. Multinomial logistic regression was used to analyse the frequency of testing utilization by the socioeconomic factors educational level, employment status, and household income, relative to the base outcome “never tested”. In order to control for possible confounding, a broad range of control variables were included: age, gender, European region of residence, country of birth, degree of urbanization, marital status, self-perceived health state, and smoking behaviour. Finally, to quantify the strength of the association, we calculated the corresponding Odds Ratios (OR) and 95% confidence intervals (95% CI).

As a subsequent analysis, we measured the extent of income-based inequality. Absolute and relative inequality indices were calculated to show whether up-to-date test utilization is more concentrated among the poorer or richer subgroups. We controlled for age, gender (colorectal) and the other explanatory variables to quantify whether the probability of screening participation is equal among income quintiles. We calculated the Slope Index of Inequality (SII) and Relative Index of inequality (RII) of cancer screening utilization by European region of residence (Regidor, 2004). These measures can be interpreted as a rate difference and rate ratio comparing those with the very lowest to those with the very highest incomes. Firstly, the previously mentioned outcome variables, were collapsed into three binary variables representing whether individuals ever had mammography (yes/no), cervical smear test (yes/no), or colorectal test (yes/no). Secondly, a Ridit score variable was computed by ranking household income and we conducted a linear regression to calculate the SII, and a Poisson regression to calculate the RII (Regidor, 2004). A positive SII-value and an RII-value greater than one indicate that the likelihood of up-to-date testing is greater among the higher income groups.

## 3 Results

### 3.1 Sample characteristics

Of the 308,246 survey participants, 56,807 (34% of the female respondents) were in the target population for breast cancer screening, 95,352 (57%) for cervical cancer screening and 125,239 (41% of all respondents) were in the target population for colorectal cancer screening (Table 1). A majority of women aged between 50 and 69 years had a mammography within the past two years (63.5%). Among women aged between 30 and 64 years, 71.3% (68,026) had their last cervical smear test within the past three years and 14.9% (14,186) were tested more than three years ago. A smaller group of women had never received mammography (12.8%, 7,281 women) or a cervical smear test (10.6%, 10,080 women). For colorectal testing 49.4% (61,881) of the respondents never had a faecal occult blood test or colonoscopy and 37.7% (47,244) of the respondents received their last FOB-test within the past two years or had a colonoscopy within the past ten years. The proportions receiving a test by region are presented in Appendix Table 2.

### 3.2 Mammography utilization

**Fig. 1:**
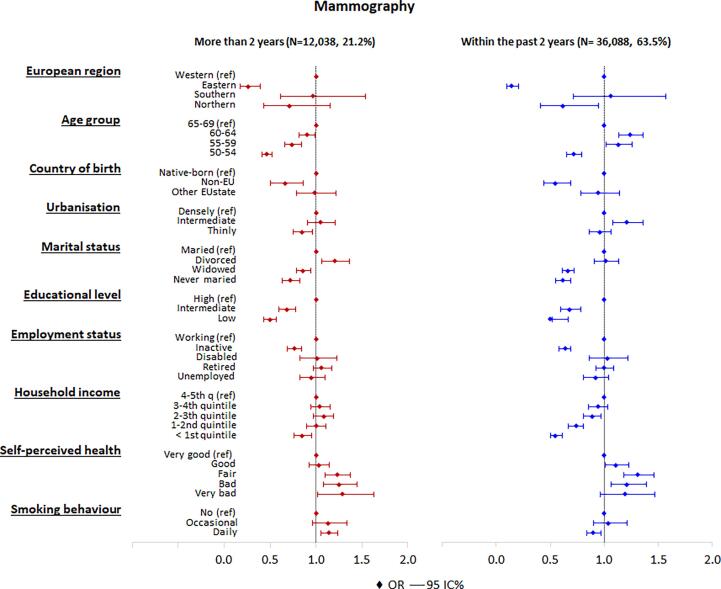
Adjusted odds ratios and 95% confidence intervals of having mammography more than two years ago and within the past two years (multivariate analysis). The base category is “never screened”.

Women living in Eastern Europe (OR = 0.14), or Northern Europe (OR = 0.62), or born in a non-EU country (OR = 0.55) were less likely than native-born women or women living in Western Europe to have had mammography in the past two years (Fig. 1). Women age 50–54 (OR = 0.72) were less likely than women age 65–69 to have had mammography in the past two years. Also never married women (OR = 0.62), widowed women (OR = 0.66), women with a low (OR = 0.50), or intermediate (OR = 0.68) educational level, and inactive women (OR = 0.64) were less likely to have had a mammogram in the past two years. There was a clear trend in household income: women with a low household income were less likely to have had mammography in the past two years (OR = 0.55) than women with a high household income. Women having a fair or bad self-perceived health (OR = 1.31 and 1.21) were more likely to have had mammography within the past two years than women with a very good self-perceived health.

In general, the same pattern was observed for women having had mammography more than two years ago versus never attending women, except of differences in the effects of age, urbanisation and smoking.

### 3.3 Cervical smear test utilization

**Fig. 2:**
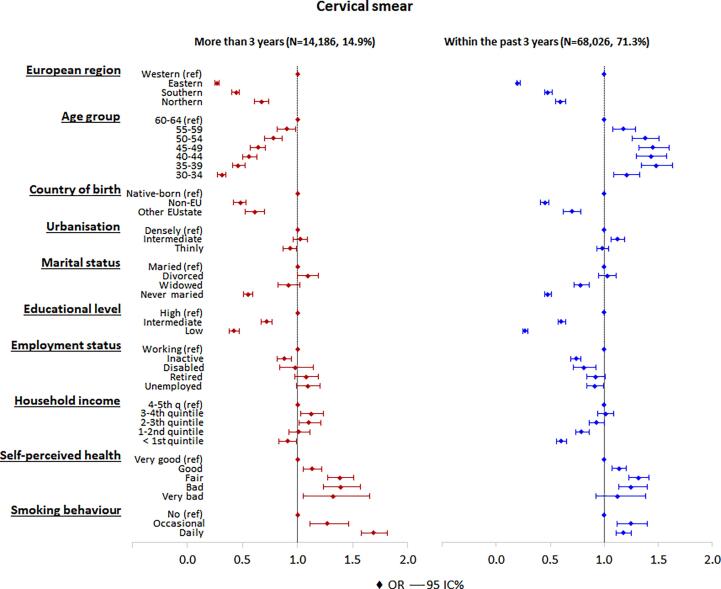
Adjusted odds ratios and 95% confidence intervals of having a cervical smear test more than three years ago and within the past three years (multivariate analysis). The base category is “never screened”.

Women living in Eastern (OR = 0.20), Southern (OR = 0.48) and Northern (OR = 0.59) Europe were less likely than women living in Western Europe to have a cervical smear in the past three years (Fig. 2). Also women born in a non-EU state (OR = 0.45) or other EU state (OR = 0.70), and widowed (OR = 0.78) or never married women (OR = 0.48) were less likely than native-born, or married women to have had a cervical smear test in the past three years. A low (OR = 0.27) or intermediate (OR = 0.60) educational level, and a low household income (OR = 0.60) were also associated with a lower likelihood of having had a cervical smear test in the past three years. Women younger than age 60–64 were more likely than women age 60–64 to have had a cervical smear in the past three years. Being a daily (OR = 1.18) or occasional smoker (OR = 1.25) increased the probability of having had a cervical cancer test or of being up to date with screening, compared to non-smokers. Overall, a similar pattern was observed when comparing the women having had a cervical smear test more than three years ago, except for age, which showed a decreasing trend for the younger ages.

### 3.4 Colorectal testing: FOB-test and colonoscopy

**Fig. 3:**
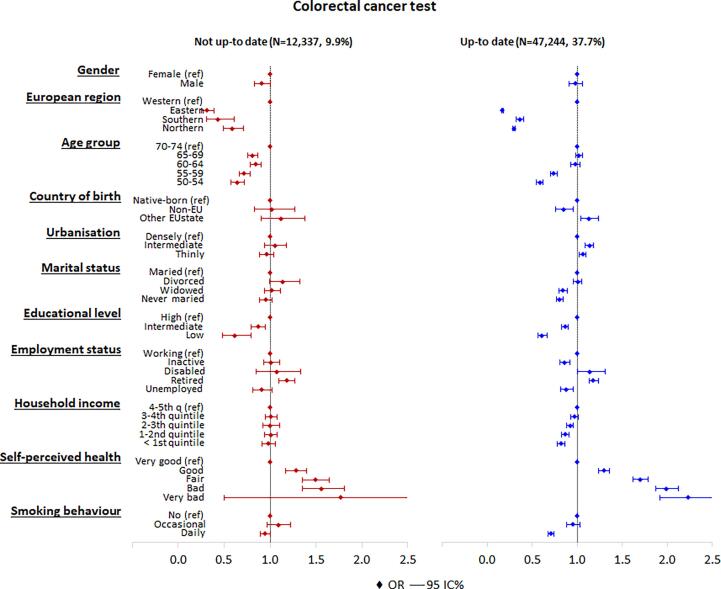
Adjusted odds ratios and 95% confidence intervals of being up-to date with colorectal cancer testing (having a FOB-test within the past 2 years or colonoscopy within the past 10 years) or being not up-to date (having a FOB-test more than 2 years ago or colonoscopy more than 10 years ago) (multivariate analysis). The base category is “never screened”.

Gender did not show a significant association with colorectal cancer screening utilization (Fig. 3). Individuals living in Eastern (OR = 0.17), Southern (OR = 0.36), or Northern Europe (OR = 0.30) were less likely to be up to date with colorectal cancer testing than individuals living in Western Europe. Individuals age 50–54 (OR = 0.59) or age 55–59 (OR = 0.74), were less likely to be up to date with colorectal cancer testing than individuals age 70–74.Likewise, individuals born in a non-EU country (OR = 0.85), were widowed (OR = 0.84) or never married (OR = 0.80) had a lower likelihood of being up to date with testing, than native born and married individuals. Having a low (OR = 0.61) or intermediate (OR = 0.87) educational level, a low household income (OR = 0.82), and being unemployed (OR = 0.88) also decreases the probability of being up to date with testing than individuals that are high educated, working and have a high household income. Finally, daily smokers, compared to non-smokers, were less likely to be tested for colorectal cancer and individuals having a fair, bad or bad self-perceived health (OR = 1.70, 1.99 and 2.23) were more likely to be up to date with testing than individuals with a very good self-perceived health. For individuals being not up to date with colorectal testing versus never attending individuals, a similar pattern was observed for differences in the effects of EU-region of residence, educational level, self-perceived health, and smoking behaviour.

### 3.5 Income-based inequality in testing utilization

The SII and RII were significant for all European regions for mammography and cervical smear test use (Table 2). The point estimates as well as the confidence intervals are positive, suggesting that women age 50–69 years within the higher income quintiles were significantly more likely to have received mammography within the past two years and women age 30–64 years within the higher income quintiles were significantly more likely to have received a cervical smear test within the past three years than their lower income counterparts. The point estimates were largest in Eastern and Southern Europe, suggesting the inequality is larger in these regions. For colorectal cancer, the SII was significant in Western and Southern Europe. The point estimate and confidence interval of the RII was above one only for colorectal cancer screening in Southern Europe, suggesting the use of up-to-date colorectal cancer testing is significantly more concentrated among the higher income groups. When we only adjusted for age (and gender in case of colorectal screening), the SII and RII comparing up-to-date colorectal test use vs not up-to-date or never tested were all significant except for colorectal cancer testing in Northern Europe (Appendix Table 3). When the ever tested respondents were compared to the never tested responders, the SII decreased and the RII was not significant in all regions for breast and cervical cancer (Appendix Table 4).

**Table 2:** Slope Index of Inequality (SII) and Relative index of inequality (RII) of testing utilization by European region of residence when comparing up-to-date vs otherwise (not up-to-date or never tested).

	SII*	p-value	95%	CI	RII*	p-value	95%	CI
*Mammography use (50*–*69 years old women)*								
Western Europe	0.138	0.000	0.112	0.163	1.194	0.000	1.114	1.279
Eastern Europe	0.110	0.000	0.076	0.144	1.244	0.000	1.116	1.385
Southern Europe	0.191	0.000	0.163	0.219	1.260	0.000	1.161	1.368
Northern Europe	0.129	0.000	0.094	0.165	1.171	0.001	1.064	1.290

*Cervical smear test use (30*–*64 years old women)*								
Western Europe	0.073	0.000	0.055	0.090	1.094	0.001	1.040	1.151
Eastern Europe	0.122	0.000	0.096	0.149	1.195	0.000	1.114	1.280
Southern Europe	0.109	0.000	0.088	0.130	1.151	0.000	1.084	1.221
Northern Europe	0.105	0.000	0.079	0.131	1.160	0.000	1.082	1.242

*Colorectal testing use (50*–*74 years old individuals)*								
Western Europe	0.045	0.008	0.013	0.077	1.051	0.122	0.986	1.121
Eastern Europe	0.009	0.456	−0.014	0.032	1.021	0.710	0.915	1.140
Southern Europe	0.161	0.000	0.134	0.189	1.487	0.000	1.353	1.636
Northern Europe	0.026	0.076	−0.003	0.055	1.053	0.355	0.944	1.176

* The following variables were included: age, gender (in case of colorectal), country of birth, degree of urbanization, marital status, educational level, employment status, self-perceived health, and smoking behaviour. A positive SII-value and an RII-value greater than one indicate that the likelihood of testing is greater among the higher income groups.

## 4 Discussion

This is the first study that evaluated socio-economic inequalities in the use of testing for three cancer sites, while differentiating whether individuals are up to date with testing, and how these inequalities varied between different European regions. The results show that, in general, factors associated with a lower likelihood of being up to date with cancer testing are: having a low or intermediate educational level, being inactive or unemployed, having a low household income, being born outside an EU country, and never being married or widowed. Contrary to what we speculated, having fair, bad or very bad health increased the probability of being up-to-date with screening. This could be a consequence of the inability in the data to distinguish screening from diagnostic investigations, with those in poor health disproportionately being investigated, but for diagnosis of suspected cancer. The factors were largely consistent over the cancer types. Furthermore, there is income-based inequality in the use of mammography and cervical smears in all European regions. This income-based inequality appeared to be the greatest in Southern and Eastern Europe, although we did not test the difference between the regions for significance and therefore the findings could be explained by chance.

Our findings are in accordance with previous studies which report that individuals within a lower socio-economic position are less likely to attend cancer screening compared to individuals within a higher socio-economic position (Deandrea et al., 2010, Hoeck et al., 2019, Willems and Bracke, 2018, Louwman et al., 2007, Douglas et al., 2016, Euler-Chelpin et al., 2008, Wardle et al., 2016). For example, ethnic minorities, rural area inhabitants, unemployed people, migrants and people living in deprived households are participating less in cancer screening (Willems and Bracke, 2018, Damiani et al., 2012, Wuebker, 2012, von Wagner et al., 2011, Pankakoski et al., 2020, Breast cancer screening programme Annual Review, 2018). There are many reasons for differences in screening participation, including differences in knowledge and attitudes, such as the value of future benefits (time preferences) and trust in the system (Deandrea et al., 2010, Hurtado et al., 2015, Gurmankin Levy et al., 2006) as well as the existence of structural and systemic barriers to access, such as costs, distance, and time (Bastos et al., 2010, Smith et al., 2019). In Southern and Eastern European countries there is more opportunistic screening, possibly leading to less participation by low social-economic groups. Also, organised screening programmes started already decades ago in Northern and Western European countries, and therefore the status of implementation is higher in these regions in comparison with the Southern and Eastern European countries, where in some cases the programme doesn’t reach the entire population. Implementing organised screening, aimed to invite the entire population in the Southern and Eastern European countries will probably reduce inequalities.

This study has several limitations. Within the second wave EHIS dataset there were no data available about the reasoning behind cancer testing. Hence, no distinction could be made between population-based screening by invitation, opportunistic testing or even the use of a test as a result of symptoms or other medical reason. In the years before the survey period (before 2013) not all countries had organized screening programmes. Especially for colorectal cancer, organised screening programmes were lacking, and therefore, overall less people were tested, which may also mask possible effects of inequality. In countries with organised screening programmes in that period, there was often a substantial amount of opportunistic testing continuing. For example, in Finland in 2010–2014, 38% of all cervical smears were taken within the programme and 62% outside the programme (Pankakoski et al., 2020). This co-existence of opportunistic and organised screening complicates an analysis by cancer screening organisation method. In addition, we defined fixed intervals of screening (2 years for breast cancer, 3 years for cervical cancer and 2 or 10 years for colorectal cancer). These intervals vary between countries with an organised screening programme. Therefore, individuals might be incorrectly classified as being up-to date with screening or not. Additionally, within the analysis we only determined differences in inequality between European regions. In some regions, countries are very heterogeneous. For example, the lower utilization of mammography in Northern Europe is caused by the low utilization in Estonia, Lithuania and Latvia, despite high screening participation in Sweden, Denmark and Finland. Another limitation is that household income was only available in European quintiles and therefore quintiles are likely to reflect differences in the income distribution between countries more than individual inequalities within countries. For example, even Southern EU citizens in the top of the regional social hierarchy will be more probably classified in the lowest EU quintiles and the SII and RII will be diluted.

Finally, this study focused on socio-economic inequality in the use of cancer testing. However, inequality can be present in each phase along the cancer care pathway, so inequalities in screening findings, survival, and in the access to cancer treatment should also be taken into account (Sarfati et al., 2010). For example, survival can vary by geographical area, which indicates the differences in access to timely cancer treatment and health services of high quality (Paper, 2017). Furthermore, deprived women with screen-detected breast cancer are more likely to face barriers like travel time and distance, inability to take time off work, and lack of health information (Bigby and Holmes, 2005, Bradley et al., 2002, Laudicella et al., 2012). An Italian study found that the breast cancer screening programme reduced disparities in the access to treatment (Zengarini et al., 2016).

Despite the existence of uniform population-based screening within a country, social subgroups tend to have different information needs due to underlying differences in for example knowledge and awareness (Hoeck et al., 2019, von Wagner et al., 2011). Therefore, it is recommended that population-based screening should be combined with strategies that are tailored to the needs of different groups within society (Hoeck et al., 2019, Spadea et al., 2010). However, comprehensive guidelines or recommendations on which strategies to use are limited to a few countries (Paper, 2017).

In conclusion, this study found that there is a persistent gap in test utilization to early detection of cancer, representing inequality related to country of birth, attained educational level, employment status, and household income. Additionally, it shows that there is income-based inequality in test utilization in all European regions, but the level of inequality is different in the four European regions. It is of great importance that the reasons for not participating in testing will be further examined, such that barriers for testing can be solved.

## Declaration of Competing Interest

The authors declare that they have no known competing financial interests or personal relationships that could have appeared to influence the work reported in this paper.
