# Supplementary material for: Socio-economic inequality of utilization of cancer testing in Europe: A cross-sectional study
Source: Prev Med Rep. 2022 Feb 8;26:101733. doi: 10.1016/j.pmedr.2022.101733 (PMC8850331; doi:10.1016/j.pmedr.2022.101733)
Supplement: Supplementary data 1 [file mmc1.docx]

**Appendix 1. Further information on SII and RII**

We measured inequality in testing utilization across European regions. We estimated the socioeconomic inequality in health using bivariate measures to calculate differences in health (testing utilization) among values of a socioeconomic variable (household income) (Regidor, 2004; Wagstaff et al., 1991; ScotPHO, 2021). We calculated the SII and RII to investigate the extent of (un)equal distribution of a health variable or the use of a health service, which is in this case screening participation, across subgroups of household income level as socioeconomic factor.

 The SII and RII take account of the values for all groups and the population size in each subgroup, based on the assumption that the data is ranked. Hence, we ranked  the variable household income. These measures can be interpreted as a rate difference (SII) and rate ratio (RII) comparing those with the very lowest to those with the very highest incomes (Regidor, 2004; Wagstaff et al., 1991; ScotPHO, 2021).

In summary:

- The Ridit score variable was created by ranking the variable Household income as fractional rank.
- Next, we added this variable in the model and we performed a linear regression to get the coefficient of the ranked variable which represents the SII (i.e. Regidor, 2004; ScotPHO, 2021).
- The RII was obtained by running a Poisson regression between the rank of "Household income" and screening participation. The log of the coefficient of the ranked variable (ridit score) provides the RII (i.e. Regidor, 2004; ScotPHO, 2021).

**References**

Wagstaff, A., Paci, P., & Van Doorslaer, E. (1991). On the measurement of inequalities in health. *Social science & medicine, 33*(5), 545-557.

Regidor, E. (2004). Measures of health inequalities: Part 2. *Journal of Epidemiology & Community Health, 58*(11), 900–903. [https://doi.org/10.1136/jech.2004.023036](https://eur01.safelinks.protection.outlook.com/?url=https%3A%2F%2Fdoi.org%2F10.1136%2Fjech.2004.023036&data=04%7C01%7Ce.heijnsdijk%40erasmusmc.nl%7C2eecb7761d7c4d9e05c708d94ab0421d%7C526638ba6af34b0fa532a1a511f4ac80%7C0%7C1%7C637622943147495105%7CUnknown%7CTWFpbGZsb3d8eyJWIjoiMC4wLjAwMDAiLCJQIjoiV2luMzIiLCJBTiI6Ik1haWwiLCJXVCI6Mn0%3D%7C1000&sdata=m%2FpH6HryVFR%2FzXZhts1EKQoei%2BL4AcXokrXRCquzJtE%3D&reserved=0)

Measuring health inequalities. The Scottish Public Health Observatory. <https://www.scotpho.org.uk/comparative-health/measuring-inequalities/>. Updated 08 June 2021. Accessed July 2021.

**For more information see also:**

Moreno-Betancur, M., Latouche, A., Menvielle, G., Kunst, A. E., & Rey, G. (2015). Relative index of inequality and slope index of inequality: a structured regression framework for estimation. *Epidemiology,26*(4), 518-527.

Conway, D.I., McMahon, A.D., Brown, D. & Leyland, A.H. (2019). [Chapter 4: Measuring socioeconomic status and inequalities](https://eur01.safelinks.protection.outlook.com/?url=http%3A%2F%2Fpublications.iarc.fr%2F_publications%2Fmedia%2Fdownload%2F5317%2F8301d5d6df552c8172158edba49bb24de652eb9d.pdf&data=04%7C01%7Ce.heijnsdijk%40erasmusmc.nl%7C2eecb7761d7c4d9e05c708d94ab0421d%7C526638ba6af34b0fa532a1a511f4ac80%7C0%7C1%7C637622943147505062%7CUnknown%7CTWFpbGZsb3d8eyJWIjoiMC4wLjAwMDAiLCJQIjoiV2luMzIiLCJBTiI6Ik1haWwiLCJXVCI6Mn0%3D%7C1000&sdata=giSenbKucCBuZ9BuEMVYOLl9nWfvZnSqpJT3mu7dLh8%3D&reserved=0). In: Vaccarella, S., Lortet-Tieulent, J., Saracci, R., Conway, D. I., Straif, K., & Wild, C. P. *Reducing social inequalities in cancer: evidence and priorities for research*. IARC Scientific Publication; 2019.

**Appendix Table 1. Characteristics of the EHIS sample if up to date with cancer testing**

|  | **Up to date ^a^ with Mammography**  **(50-69 aged females)**  **N=36,088** | **Up to date ^a^ with Cervical smear testing**  **(30-64 aged females)**  **N= 68,026** | **Up to date ^a^ with Colorectal testing**  **(age 50-74)**  **N=47,244** |
| --- | --- | --- | --- |
|  |  |  | ***Males:*** *21,642 (45.8%)*  ***Females:*** *25,602 (54.2%)* |

| **Variable** | **N** | **%** |  |  | **N** | **%** |  | **N** | **%** |
| --- | --- | --- | --- | --- | --- | --- | --- | --- | --- |
| ***Age*** |  |  |  |  |  |  |  |  |  |
| 30-34 |  |  |  |  | 8496 | 12.5 |  |  |  |
| 35-39 |  |  |  |  | 9800 | 14.4 |  |  |  |
| 40-44 |  |  |  |  | 10497 | 15.4 |  |  |  |
| 45-49 |  |  |  |  | 10898 | 16.0 |  |  |  |
| 50-54 | 9330 | 25.9 |  |  | 10498 | 15.4 |  | 8283 | 17.5 |
| 55-59 | 9603 | 26.6 |  |  | 9475 | 13.9 |  | 9108 | 19.3 |
| 60-64 | 9234 | 25.6 |  |  | 8362 | 12.3 |  | 10691 | 22.6 |
| 65-69 | 7921 | 21.9 |  |  |  |  |  | 10803 | 22.9 |
| 70-74 |  |  |  |  |  |  |  | 8359 | 17.7 |
| ***European region of residence* ^b^** |  |  |  |  |  |  |  |  |  |
| Western Europe | 12590 | 34.9 |  |  | 23504 | 34.6 |  | 22307 | 47.2 |
| Eastern Europe | 6056 | 16.8 |  |  | 13422 | 19.7 |  | 6643 | 14.1 |
| Southern Europe | 10640 | 29.5 |  |  | 18655 | 27.4 |  | 11380 | 24.1 |
| Northern Europe | 6802 | 18.8 |  |  | 12445 | 18.3 |  | 6914 | 14.6 |
| ***Country of birth*** |  |  |  |  |  |  |  |  |  |
| Native-born | 33117 | 91.8 |  |  | 60904 | 89.5 |  | 43244 | 91.5 |
| Non-EU country | 1528 | 4.2 |  |  | 3695 | 5.4 |  | 1823 | 3.9 |
| Other EU state | 1151 | 3.2 |  |  | 2897 | 4.3 |  | 1672 | 3.5 |
| Missing | 292 | 0.8 |  |  | 530 | 0.8 |  | 505 | 1.1 |
| ***Urbanization*** |  |  |  |  |  |  |  |  |  |
| Densely-populated | 12414 | 34.4 |  |  | 23757 | 34.9 |  | 16037 | 33.9 |
| Intermediate-populated | 11243 | 31.2 |  |  | 21067 | 31.0 |  | 14995 | 31.7 |
| Thinly-populated | 12400 | 34.4 |  |  | 23142 | 34.0 |  | 16151 | 34.2 |
| Missing | 31 | 0.1 |  |  | 60 | 0.1 |  | 61 | 0.1 |
| ***Marital status*** |  |  |  |  |  |  |  |  |  |
| Married | 24502 | 67.9 |  |  | 45666 | 67.1 |  | 33245 | 70.4 |
| Divorced | 4675 | 13.0 |  |  | 7817 | 11.5 |  | 5466 | 11.6 |
| Widowed | 3913 | 10.8 |  |  | 2932 | 4.3 |  | 4666 | 9.9 |
| Never married | 2924 | 8.1 |  |  | 11472 | 16.9 |  | 3711 | 7.9 |
| Missing | 74 | 0.2 |  |  | 139 | 0.2 |  | 156 | 0.3 |
| ***Educational level*** |  |  |  |  |  |  |  |  |  |
| High | 8736 | 24.2 |  |  | 24255 | 35.7 |  | 11833 | 25.0 |
| Intermediate | 21510 | 59.6 |  |  | 39336 | 57.8 |  | 28486 | 60.3 |
| Low | 5635 | 15.6 |  |  | 4100 | 6.0 |  | 6647 | 14.1 |
| Missing | 207 | 0.6 |  |  | 335 | 0.5 |  | 278 | 0.6 |
| ***Employment status*** |  |  |  |  |  |  |  |  |  |
| Working | 15729 | 43.6 |  |  | 45172 | 66.4 |  | 17071 | 36.1 |
| Other | 4972 | 13.8 |  |  | 10082 | 14.8 |  | 3498 | 7.4 |
| Permanently disabled | 1422 | 3.9 |  |  | 1767 | 2.6 |  | 1813 | 3.8 |
| In (early) retirement | 11935 | 33.1 |  |  | 5520 | 8.1 |  | 22888 | 48.4 |
| Unemployed | 1785 | 4.9 |  |  | 5067 | 7.4 |  | 1728 | 3.7 |
| Missing | 245 | 0.7 |  |  | 418 | 0.6 |  | 246 | 0.5 |
| ***Household monthly income quintiles*** |  |  |  |  |  |  |  |  |  |
| Between 4th-5th quintile | 7874 | 21.8 |  |  | 15450 | 22.7 |  | 10288 | 21.8 |
| Between 3rd-4th quintile | 7405 | 20.5 |  |  | 14639 | 21.5 |  | 9693 | 20.5 |
| Between 2nd-3rd quintile | 6929 | 19.2 |  |  | 12556 | 18.5 |  | 9288 | 19.7 |
| Between 1st-2nd quintile | 6241 | 17.3 |  |  | 10924 | 16.1 |  | 8561 | 18.1 |
| Below 1st quintile | 5209 | 14.4 |  |  | 10180 | 15.0 |  | 6884 | 14.6 |
| Missing | 2430 | 6.7 |  |  | 4277 | 6.3 |  | 2530 | 5.4 |
| ***Self-perceived health*** |  |  |  |  |  |  |  |  |  |
| Very good | 5009 | 13.9 |  |  | 15683 | 23.1 |  | 5697 | 12.1 |
| Good | 15685 | 43.5 |  |  | 33512 | 49.3 |  | 19742 | 41.8 |
| Fair | 11678 | 32.4 |  |  | 14851 | 21.8 |  | 15917 | 33.7 |
| Bad | 2934 | 8.1 |  |  | 3190 | 4.7 |  | 4596 | 9.7 |
| Very Bad | 665 | 1.8 |  |  | 634 | 0.9 |  | 1127 | 2.4 |
| Missing | 117 | 0.3 |  |  | 156 | 0.2 |  | 165 | 0.3 |
| ***Smoking behaviour*** |  |  |  |  |  |  |  |  |  |
| No smoking | 29262 | 81.1 |  |  | 52371 | 77.0 |  | 38725 | 82.0 |
| Occasional smoking | 1136 | 3.1 |  |  | 3099 | 4.6 |  | 1489 | 3.2 |
| Daily smoking | 5428 | 15.0 |  |  | 12218 | 18.0 |  | 6662 | 14.1 |
| Missing | 262 | 0.7 |  |  | 338 | 0.5 |  | 368 | 0.8 |

**a. Up to date= received mammography within past 2 years, cervical smear test within past 3 years, or received FOB-test within past two years and/or received colonoscopy within past ten years**

**b. Northern Europe: Denmark, Estonia, Finland, Ireland, Lithuania, Latvia, Norway, Sweden**

**Western Europe: Austria, Belgium, Germany, France, Luxembourg, Netherlands, United Kingdom**

**Eastern Europe: Bulgaria, Czech Republic, Hungary, Poland, Romania,** **Slovenia, Slovakia**

**Southern Europe: Cyprus,** **Greece, Spain,** **Croatia, Italy, Portugal**

|  |
| --- |

**Table 2. Proportions of up-to date, not up-to-date and never tested by European region**

|  | Western | Eastern | Southern | Northern |
| --- | --- | --- | --- | --- |
| Breast |  |  |  |  |
| Up-to-date | 69.4 | 45.2 | 71.5 | 65.5 |
| Not up-to-date | 20.1 | 23.9 | 19.2 | 22.5 |
| Never tested | 6.4 | 28.8 | 7.9 | 10.3 |
| Missing | 4.0 | 2.1 | 1.4 | 1.7 |
|  |  |  |  |  |
| Cervical |  |  |  |  |
| Up-to-date | 75.2 | 62.1 | 72.5 | 74.2 |
| Not up-to-date | 14.2 | 16.5 | 13.7 | 15.8 |
| Never tested | 5.0 | 18.7 | 12.2 | 8.0 |
| Missing | 4.8 | 2.6 | 1.6 | 2.0 |
| Not applicable | 0.8 |  |  |  |
|  |  |  |  |  |
| Colorectal |  |  |  |  |
| Up-to-date | 55.3 | 22.9 | 34.4 | 30.3 |
| Not up-to-date | 10.6 | 8.3 | 8.5 | 12.4 |
| Never tested | 29.6 | 65.8 | 55.8 | 54.3 |
| Missing | 4.4 | 3.0 | 1.3 | 3.1 |

**Table 3. Slope Index of Inequality (SII) and Relative index of inequality (RII) of testing utilization by European region of residence, controlled on age (and gender in case of colorectal cancer) only.**

| **European region of residence** | **SII** | **p-value** | **95%** | **CI** | **RII** | **p-value** | **95%** | **CI** |
| --- | --- | --- | --- | --- | --- | --- | --- | --- |
| ***Mammography use***  ***(50-69 years old women)*** |  |  |  |  |  |  |  |  |
| Western Europe | **0.135** | **0.000** | **0.111** | **0.159** | **1.204** | **0.000** | **1.130** | **1.283** |
| Eastern Europe | **0.155** | **0.000** | **0.123** | **0.188** | **1.398** | **0.000** | **1.273** | **1.534** |
| Southern Europe | **0.191** | **0.000** | **0.163** | **0.219** | **1.298** | **0.000** | **1.212** | **1.391** |
| Northern Europe | **0.199** | **0.000** | **0.234** | **0.127** | **1.330** | **0.000** | **1.218** | **1.451** |
| ***Cervical smear test use***  ***(30-64 years old women)*** |  |  |  |  |  |  |  |  |
| Western Europe | **0.113** | **0.000** | **0.097** | **0.129** | 1.151 | 0.000 | 1.101 | 1.206 |
| Eastern Europe | **0.222** | **0.000** | **0.199** | **0.245** | **1.415** | **0.000** | **1.332** | **1.502** |
| Southern Europe | **0.212** | **0.000** | **0.193** | **0.230** | **1.340** | **0.000** | **1.276** | **1.409** |
| Northern Europe | **0.156** | **0.000** | **0.132** | **0.180** | **1.228** | **0.000** | **1.153** | **1.309** |
| ***Colorectal testing use***  ***(50-74 years old individuals)*** |  |  |  |  |  |  |  |  |
| Western Europe | **0.058** | **0.000** | **0.030** | **0.085** | **1.097** | **0.004** | **1.030** | **1.169** |
| Eastern Europe | **0.051** | **0.000** | **0.030** | **0.072** | **1.220** | **0.000** | **1.102** | **1.351** |
| Southern Europe | **0.148** | **0.000** | **0.123** | **0.172** | **1.484** | **0.000** | **1.366** | **1.611** |
| Northern Europe | 0.028 | 0.070 | -0.002 | 0.058 | 1.078 | 0.171 | 0.968 | 1.201 |

**Table 4. Slope Index of Inequality (SII) and Relative index of inequality (RII) of testing utilization by European region of residence, when comparing ever tested vs never tested.**

| **European region of residence** | **SII*** | **p-value** | **95%** | **CI** | **RII*** | **p-value** | **95%** | **CI** |
| --- | --- | --- | --- | --- | --- | --- | --- | --- |
| ***Mammography use***  ***(50-69 years old women)*** |  |  |  |  |  |  |  |  |
| Western Europe | **0.045** | **0.000** | **0.029** | **0.062** | 1.022 | 0.308 | 0.980 | 1.066 |
| Eastern Europe | **0.088** | **0.000** | **0.056** | **0.120** | 1.041 | 0.153 | 0.985 | 1.099 |
| Southern Europe | **0.093** | **0.000** | **0.074** | **0.112** | 1.045 | 0.075 | 0.996 | 1.096 |
| Northern Europe | **0.059** | **0.000** | **0.034** | **0.084** | 1.026 | 0.365 | 0.970 | 1.085 |
| ***Cervical smear test use***  ***(30-64 years old women)*** |  |  |  |  |  |  |  |  |
| Western Europe | **0.030** | **0.000** | **0.020** | **0.040** | 1.015 | 0.361 | 0.983 | 1.049 |
| Eastern Europe | **0.078** | **0.000** | **0.057** | **0.099** | 1.039 | 0.070 | 0.997 | 1.082 |
| Southern Europe | **0.057** | **0.000** | **0.040** | **0.074** | 1.029 | 0.113 | 0.991 | 1.068 |
| Northern Europe | **0.053** | **0.000** | **0.037** | **0.070** | 1.030 | 0.167 | 0.987 | 1.076 |
| ***Colorectal testing use***  ***(50-74 years old individuals)*** |  |  |  |  |  |  |  |  |
| Western Europe | **0.034** | **0.001** | **0.014** | **0.055** | 1.011 | 0.542 | 0.977 | 1.045 |
| Eastern Europe | -0.007 | 0.606 | -0.032 | 0.019 | 0.994 | 0.798 | 0.948 | 1.042 |
| Southern Europe | **0.168** | **0.000** | **0.141** | **0.195** | **1.112** | **0.000** | **1.066** | **1.161** |
| Northern Europe | -0.002 | 0.913 | -0.033 | 0.030 | 0.995 | 0.842 | 0.946 | 1.046 |
